# The effect of cold water intake on heart rate variability in young women: the co-activation of the sympathetic and parasympathetic branches of the autonomic nervous system

**DOI:** 10.3389/fphys.2025.1627110

**Published:** 2025-09-18

**Authors:** Isidora Knežević, Borislav Tapavički, Vuk Vukosavljević, Nemanja Maletin, David Ivanov, Milica Vuletić, Dragan Burić, Otto Barak

**Affiliations:** ^1^ Department of Physiology, Faculty of Medicine, University of Novi Sad, Novi Sad, Serbia; ^2^ Department of Pathophysiology and Laboratory Medicine, Faculty of Medicine, University of Novi Sad, Novi Sad, Serbia; ^3^ Center for Laboratory Medicine, University Clinical Center of Vojvodina, Novi Sad, Serbia

**Keywords:** heart rate variability, cold water intake, autonomic nervous system, luteal phase, young females

## Abstract

**Background:**

Heart rate variability (HRV) reflects the autonomic nervous system’s (ANS) influence on heart rate control. Daily essential activities, such as water ingestion, affect HRV. This study aims to investigate the effect of cold water intake on HRV in young, healthy females.

**Materials and Methods:**

Fourteen healthy young females participated in the study during the luteal phase of their menstrual cycle. R-R intervals were recorded using an ECG, 5 min before and 35 min after the ingestion. We used *LabChart* to determine HRV parameters from time (HR, rMSSD, pRR50) and frequency (LF, HF) domains. Data were processed in *JASP* using repeated-measures *ANOVA* to compare parameters across five different 5-min ECG segments. A *post hoc Bonferroni* test revealed specific time points where significant differences occurred.

**Results:**

Statistically significant differences were found in all HRV parameters. The *post hoc* test revealed differences between pre-ingestion and post-ingestion intervals, while no significant differences were found among the post-ingestion periods. Both branches of the ANS were activated. A statistically significant increase in LF, indicating sympathetic activation, and in rMSSD and pRR50 components, reflecting parasympathetic activity, was observed immediately after cold water ingestion compared to the pre-ingestion period. HF became statistically significantly higher 10 min after cold water ingestion.

**Conclusion:**

In young healthy females, the ANS responds coordinately to cold water ingestion, activating both branches to maintain homeostasis, preventing heat loss through sympathetic activation and limiting excessive increases in blood pressure and heart rate via parasympathetic regulation.

## 1 Introduction

The autonomic nervous system (ANS) plays a crucial role in regulating heart rate (HR), a dynamic and fluctuating value, reflecting healthy regulatory mechanisms capable of adapting to constantly changing environment (Pham et al., 2021). Heart rate variability (HRV) is defined as the variation in time intervals between consecutive R-waves on an ECG, which occur even at rest (Li et al., 2019). These variations result from the continuous modulation of the heart by the ANS to maintain homeostasis and respond to changing physiological demands (Lundell and Ojanen, 2023). Therefore, measuring HRV provides a non-invasive tool for assessing the balance between sympathetic and parasympathetic influences on heart function (Hayano and Yuda, 2021). In addition to the traditional factors such as respiratory rate, blood pressure, and neuro-endocrine function, water ingestion, a routine and essential daily activity, has recently become the focus of research exploring its effects on ANS (Lundell and Ojanen, 2023; Pereira et al., 2017; Tiwari et al., 2021).

Several studies investigated the hemodynamic and autonomic effects of cold-water intake (Pereira et al., 2017; Girona et al., 2014; Routledge et al., 2002; Jordan et al., 1999). Ingestion of cold water induces a transient shift between sympathetic and parasympathetic dominance, suggesting that it may modulate cardiovascular control mechanisms through thermosensitive autonomic reflexes. Despite its apparent simplicity, this physiological response involves complex neural pathways integrating inputs from thermoreceptors in the oral cavity, esophagus, and stomach (Siddangoudra and Arjunwadekar, 2017). The physiological response to cold water ingestion in young, healthy individuals includes peripheral vasoconstriction, leading to a rise in blood pressure caused by a brief dominance of the sympathetic nervous system. Vagally mediated bradycardia follows, aimed to prevent an excessive increase in blood pressure and heart rate (Siddangoudra and Arjunwadekar, 2017; Arjunwadekar and Siddangoudra, 2019). However, the clinical significance of these acute autonomic responses remains unclear, prompting further investigation into their effects, particularly through the use of heart rate variability.

Restricting ice fluids was previously a standard of care for patients after acute myocardial infarction due to concerns that intake of ice fluids could trigger coronary vasospasm and increase myocardial workload (Pereira et al., 2017; Siddangoudra and Arjunwadekar, 2017). However, more recent studies have questioned these assumptions, suggesting that cold water ingestion may not be as harmful as once believed (Siddangoudra and Arjunwadekar, 2017). Moreover, some studies have shown that acute ingestion of cold water in healthy individuals induces predictable and transient changes in autonomic tone, with the potential to influence cardiovascular stability (Tiwari et al., 2021). This shift in understanding has prompted further exploration of how the ANS responds to cold water in healthy individuals, particularly in terms of HRV. Our study aims to further explore these effects, specifically focusing on the ANS response to cold water ingestion in young females during the luteal phase of the menstrual cycle, using HRV as a tool for evaluation of the ANS’s activity. By focusing on this specific phase, we aim to provide a clearer understanding of how cold water ingestion modulates autonomic function and its potential impact on cardiovascular regulation, thus eliminating the effect of hormonal fluctuations during the different phases of the menstrual cycle.

## 2 Materials and methods

### 2.1 Study design and participants

The study was conducted at the Laboratory for Functional Testing, Department of Physiology, Faculty of Medicine, University of Novi Sad, Serbia, from December 2023 to January 2024. A total of fourteen young (age 22 ± 2 years; body weight 60.42 ± 8.49 kg; height 170.14 ± 4.64 cm), healthy females participated voluntarily in the study. All participants underwent an interview regarding their disease history, had their blood pressure measured and electrocardiogram (ECG) done. Studies have shown that sympathetic activity is more dominant during the luteal phase (Yazar and Yazıcı, 2016; Sato et al., 1995; Vishrutha et al., 2012), while vagal activity predominates during the follicular phase (Vishrutha et al., 2012; Chung and Yang, 2011). Therefore, when evaluating HRV parameters in women of reproductive age, it is important to consider the hormonal influence based on the phase of the menstrual cycle at the time of assessment. The luteal phase was confirmed based on the information obtained from mobile applications in which the participants recorded the course of their menstrual cycle. The recording was performed on day 21 (±2 days) from the start of the previous menstrual period (Blagrove et al., 2020). The participants had regular menstrual cycles, with menstrual bleeding occurring regularly for at least the last 6 months and the cycle lasting between 26 and 34 days (Sato et al., 1995). Exclusion criteria included any cardiovascular, neurological or endocrine disorders, the use of oral contraceptives or any medication that can influence cardiac and autonomic function, pregnancy and the follicular phase of the menstrual cycle at the time of testing.

### 2.2 Testing procedure

All measurements were performed in the morning hours (from 8.00 a.m. to noon). On the day of testing, participants refrained from exercise, and did not consume caffeine. They were instructed to refrain from eating or drinking for at least 2 hours prior to testing to prevent postprandial autonomic influences. They were also advised to maintain a regular sleep schedule the night before the test to minimize circadian variations.

Testing was conducted in a quiet, dark room at a room temperature of 24 C. Participants remained in a supine position with their arms relaxed at their sides and legs uncrossed. Spontaneous breathing was maintained throughout the recording period, without any verbal or physical distractions. After a 10-min accommodation period, a 5-min recording of R-R intervals was performed using a single-lead electrocardiogram (ECG), with gel applied to the electrodes to improve electrical signal conduction. The positive electrode was placed under the left clavicle, and the negative electrode was placed at the same position on the right side. The third electrode, serving as a ground, was placed on the abdomen, in a vertical line with the positive electrode. The PowerLab device (ADI Instruments, New Zealand) was used for recording. After the first recording, participants consumed the entire 500 mL within 5 min, taking small, uniform sips at a steady pace without interruptions. The water temperature (4 °C) was measured with a laboratory thermometer and served in a standardized container.

### 2.3 Data analysis

After the R-waves were identified by the continuous monitoring software LabChart, all R-R intervals were visually inspected to exclude any ectopic beats and/or detect arrhythmias or artifacts. Ectopic beats were deleted, along with the post-ectopic beat, and automatically merged with adjacent R-R intervals. To ensure that only normal R-R intervals were included in the analysis and that further visual processing was performed to obtain quality and relevant data, the widely accepted term N-N intervals (normal-to-normal intervals) was used (Blagrove et al., 2020).

A total of five 5-min segments from the recording were selected for HRV analysis: the first segment was a baseline measurement, immediately before water ingestion, and the remaining four segments were taken after the participants drank the water, at the following intervals: from 0 to 5 min (point 0); 10–15 min (point 10); 20–25 min (point 20); and 30–35 min (point 30).

HRV evaluation included the linear method with the determination of parameters in both the time and frequency domains for each of the five segments, using LabChart software. The linear method of HRV analysis is excellent for short-term spectral analysis and provides insight into the current changes in the HRV spectral profile (Li et al., 2019). Statistical methods of time-domain analysis involve the mathematical quantification of the variability degree in the time intervals between successive R-waves (R-R intervals) of normal QRS complexes in continuous ECG monitoring (Sammito et al., 2024). The parameters that were determined from the time domain included average heart rate (Average Heart Rate - HR) defined as the number of heart beats per minute (bpm), root Mean Square of Successive Differences (rMSSD) assessed by taking the square root of the average of the squared differences between successive R-R intervals (ms), and the percentage of adjacent RR intervals that differ by more than 50 m (pRR50%), which is calculated by dividing the number of pairs of adjacent RR intervals differing by more than 50 m in the entire recording with the total number of all RR intervals (%) (Malik, 1996). From the frequency domain, power in low frequencies (LF) and high frequencies (HF) was determined (ms2). Each frequency band reflects different physiological processes and regulatory mechanisms (Sammito et al., 2024). The HF (0.15–0.40 Hz) or respiratory band depends on breathing and is considered to reflect the parasympathetic component, specifically the influence of the vagus nerve on cardiovascular function (Rastović et al., 2019). The LF band (0.04–0.15 Hz) reflects the activity of both components of the autonomic nervous system (Sammito et al., 2024), with some authors emphasizing the significance of predominantly sympathetic influences on LF values (Shaffer and Ginsberg, 2017). LF and HF values were log-transformed using the natural logarithm (lnLF and lnHF) to ensure normal distribution and reduce variability across participants.

### 2.4 Ethical considerations

The study protocol was approved by the Ethics Committee of the Faculty of Medicine, University of Novi Sad (approval number: 01-39/167/1). All the participants signed an informed consent form prior to testing. Participation in this study was voluntary and anonymous, with no material compensation provided.

### 2.5 Statistical analysis

Statistical processing of the obtained data was performed using the JASP version 0.18.3.0 (2022). We used the Kolmogorov-Smirnov test to assess the normality of data distribution, ensuring the appropriate application of parametric statistical analyses. A repeated-measures ANOVA was conducted to assess changes in HRV parameters across different time points. Mauchly’s test of sphericity was used to evaluate the assumption of equal variances in repeated-measures ANOVA. When the assumption of sphericity was violated, the Greenhouse-Geisser correction was applied. Initially, a global comparison of all parameters across the selected time intervals (baseline, 0, 10, 20, and 30 min) was performed, with statistical significance set at p < 0.05. If a significant main effect was found, *post hoc* Bonferroni-adjusted pairwise comparisons were conducted to determine specific time points where significant differences occurred. All the values of HRV parameters were expressed as mean ± SD.

## 3 Results

### 3.1 Results of repeated-measures ANOVA

The statistical analysis showed that for both time- and frequency-domain parameters, there is a statistically significant difference between the measured 5-min segments (HR (p = 0.02), rMSSD (p = 0.001), pRR50 (p = 0.011), LF (p = 0.002), HF (p = 0.012)).

### 3.2 Results of time domain analysis

**FIGURE 1:**
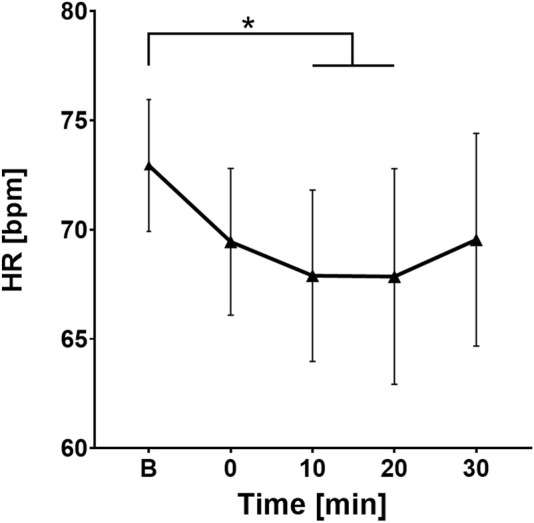
Change in heart rate values over time. B, Baseline (before cold water ingestion); 0, 0–5 min after water ingestion; 10, 10–15 min after water ingestion; 20, 20–25 min after water ingestion; 30, 30–35 min after water ingestion. *, p < 0.05. This figure shows a statistically significant decrease in HR values 10 and 20 min after intake of cold water compared to baseline.

After determining a statistically significant difference in heart rate values across different 5-min segments of the HRV recordings, the *post hoc* Bonferroni test clarified that the difference exists between the basal HR and the values at the point 10 (p = 0.03) and point 20 (p = 0.03) minute. Figure 1 shows a decrease in HR after ingesting cold water compared to the baseline values (73 ± 5 bpm), starting at the point 0 and lasting until the point 20 (69 ± 6 bpm; 68 ± 6 bpm; 68 ± 8 bpm respectively), followed by a gradual increase (70 ± 8 bpm) toward baseline values in the last 10 min.

**FIGURE 2:**
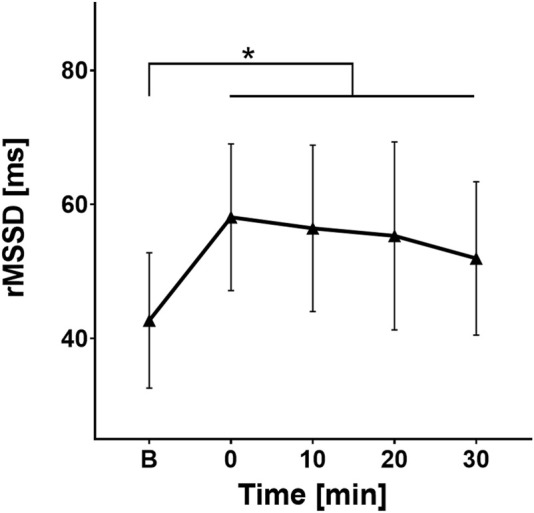
Change in rMSSD values over time. B, Baseline (before cold water ingestion); 0, 0–5 min after water ingestion; 10, 10–15 min after water ingestion; 20, 20–25 min after water ingestion; 30, 30–35 min after water ingestion. *, p < 0.05. A statistically significant increase in rMSSD values was observed at 0, 10, 20, and 30 min after cold water intake, compared to baseline values.

For rMSSD, a key parameter of parasympathetic activity, *post hoc* testing showed significant differences between baseline and the values at the points 0 (p < 0.01), 10 (p < 0.01), 20 (p < 0.01), and 30 (p = 0.028). A sharp increase in rMSSD values was observed in the first few minutes after ingesting cold water (average baseline value was 42.69 ± 16.84 m compared to the point 0 where average value was 58.08 ± 18.24 m), followed by a gradual decline over the next 30 min (56.44 ± 20.71 m; 55.3 ± 23.41 m; 51.94 ± 19.11 m respectively), showing the engagement of the parasympathetic branch of the ANS not only during the initial minutes after cold water intake, but for the next 30 min as well (Figure 2).

**FIGURE 3:**
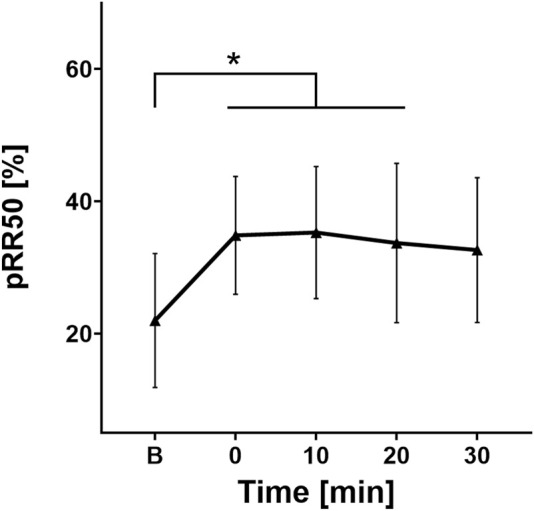
Change in pRR50 values over time. B, Baseline (before cold water ingestion); 0, 0–5 min after water ingestion; 10, 10–15 min after water ingestion; 20, 20–25 min after water ingestion; 30, 30–35 min after water ingestion. *, p < 0.05. This figure shows a statistically significant increase in pRR50 values at 0, 10, and 20 min after cold water ingestion compared to the baseline.

Similarly, for pRR50(%) the *post hoc* test confirmed significant differences between the pre-ingestion period and the point 0 (p = 0.015), 10 (p = 0.01), and 20 (p = 0.04) after ingestion, with no significant differences between the other time segments. The trend for pRR50% is similar to rMSSD, but while rMSSD peaks immediately after water ingestion, pRR50% peaks at the point 10 (35.3% ± 16.64%) and then gradually decreases toward baseline values (Figure 3).

### 3.3 Results of frequency domain analysis

The *post hoc* Bonferroni test showed a statistically significant difference between the baseline lnLF value and the lnLF values at each time point after drinking cold water (point 0 (p = 0.012), point 10 (p = 0.002), point 20 (p = 0.002), and point 30 (p = 0.001) minutes). On the other hand, no statistically significant difference was found when comparing lnLF values at different time intervals after water ingestion.

**FIGURE 4:**
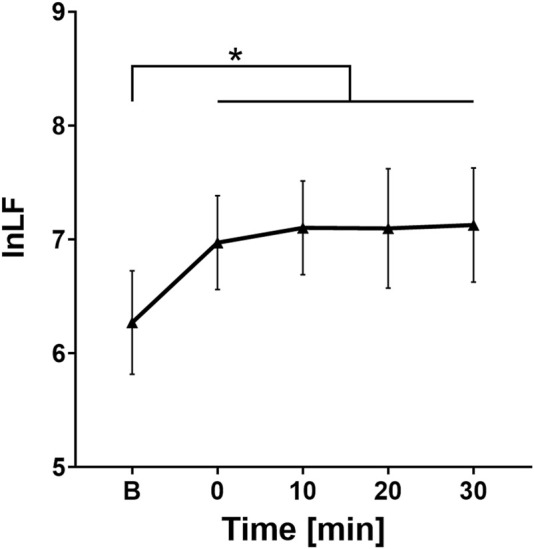
Change in lnLF values over time. B, Baseline (before cold water ingestion); 0, 0–5 min after water ingestion; 10, 10–15 min after water ingestion; 20, 20–25 min after water ingestion; 30, 30–35 min after water ingestion. *, p < 0.05. A statistically significant increase in LF values was observed at 0, 10, 20, and 30 min after cold water intake, compared to baseline values.

Figure 4 shows that the ingestion of cold water resulted in an increase in the average lnLF value compared to the baseline (6.27 ± 0.76), especially at point 0 (6.97 ± 0.68), followed by an additional increase at point 10 (7.1 ± 0.68). The values remained nearly constant and high for the next 20 min (7.09 ± 0.87; 7.12 ± 0.84, respectively).

In contrast to the LF analysis, the *post hoc* Bonferroni test for lnHF demonstrated a relevant statistical difference between the baseline lnHF value and the lnHF values at points 10 (p = 0.005), 20 (p = 0.005), and 30 (p = 0.021), but no difference was found between the baseline lnHF and HF immediately after water ingestion (at point 0). Similar to lnLF, the variability analysis of lnHF across different time intervals after fluid intake did not show any differences that could be considered statistically significant.

**FIGURE 5:**
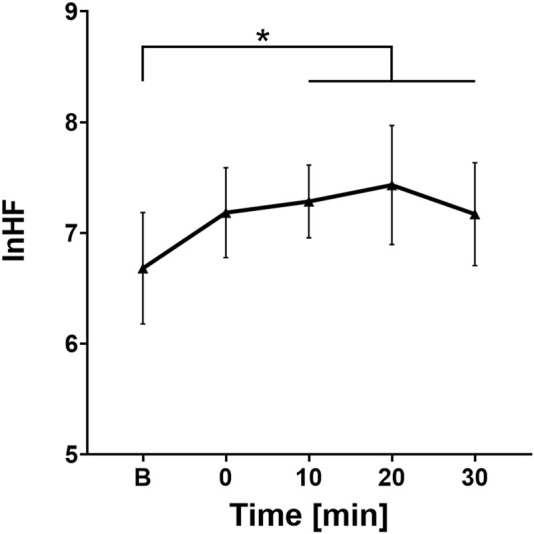
Change in lnHF values over time. B, Baseline (before cold water ingestion); 0, 0–5 min after water ingestion; 10, 10–15 min after water ingestion; 20, 20–25 min after water ingestion; 30, 30–35 min after water ingestion. *, p < 0.05. This figure illustrates a statistically significant increase in HF values at 10, 20, and 30 min following cold water ingestion, compared to baseline.

An increase in the average lnHF value followed immediately after water ingestion (average baseline value was 6.68 ± 0.84 compared to 7.18 ± 0.67 at point 0), with a gradual further rise, reaching its peak at point 20 (7.43 ± 0.89). In the last 10 min of measurement, a downward trend in lnHF values was observed (7.17 ± 0.8) (Figure 5).

## 4 Discussion

This study demonstrates that cold water ingestion elicits a rapid and coordinated autonomic response characterized by the activation of both sympathetic and parasympathetic branches of the autonomic nervous system. Evidence of sympathetic activation is seen in the immediate and sustained increase in LF power, while parasympathetic activation is reflected in the significant rise in rMSSD and pRR50 observed immediately after ingestion. Although HF power, a frequency-domain marker of parasympathetic activity, did not show significant change at the 0-min mark, it became significantly elevated from 10 min onward. This apparent delay in HF response may be due to its reliance on stable respiratory patterns and longer data windows for accurate spectral analysis, rather than a true delay in physiological vagal activation. Importantly, heart rate decreased immediately following cold water ingestion and remained suppressed for up to 20 min, despite concurrent sympathetic activation. This suggests that parasympathetic influence was strong enough to override the expected sympathetic chronotropic effect, highlighting a dynamic interplay between both branches of the ANS. Together, these findings support the presence of early autonomic coactivation followed by sustained parasympathetic dominance, underscoring the complex cardiovascular adjustments to a common physiological stimulus.

The effect of ingesting water at extreme temperatures, whether very low or high, has been a subject of numerous scientific debates, as evidenced by the many conflicting results of studies (Pereira et al., 2017). Given the intricate interplay between the sympathetic and parasympathetic systems in maintaining cardiovascular homeostasis, it is of great importance to investigate whether acute ingestion of cold water can produce significant, short-term effects on autonomic regulation and cardiovascular function. Understanding these effects may provide valuable insights into the physiological mechanisms underlying the ANS’s ability to adapt rapidly to environmental changes. Furthermore, since HRV is a reliable marker of autonomic regulation, assessing HRV parameters following cold water ingestion can help elucidate how the ANS dynamically responds to thermal and mechanical stimuli within the gastrointestinal tract.

Considering the main findings of our study, it was observed that the intake of cold water in healthy subjects has a clear modulatory effect on the sympathovagal balance. We found that the co-activation of the sympathetic and parasympathetic branches characterizes the initial response of the ANS to cold water ingestion. This is supported by the statistically significant increase in LF, rMSSD and pRR50 components observed immediately after the ingestion, compared to the pre-ingestion period. Some authors suggest that cold water stimulates nociceptive pathways in the oral cavity, which may trigger sympathetic responses (Kubota et al., 2022). The goal of this reaction is to preserve the body’s temperature and prevent significant thermal fluctuations, achieved through dermal vasoconstriction and the stimulation of metabolic processes that generate heat. Baroreceptors detect an increase in blood pressure caused by the sympathetic branch of the ANS, and, along with thermosensitive vagal nerve fibers located in the mucosa of the mouth, esophagus, and stomach, they generate nerve impulses that influence the medulla oblongata to activate the parasympathetic branch. This response leads to a decrease in heart rate and helps avoid a dramatic spike in blood pressure. Investigating the effect of cold water intake on HRV in healthy young females during the luteal phase of the menstrual cycle, we found that changes occur in both the time-domain and frequency-domain parameters.

The simplest parameter that provides insight into the balance between the two branches of autonomic control of heart function is heart rate. Based on the results obtained by comparing the heart rate before and after ingesting cold water, we observed that cold water ingestion affects the rate of impulse generation in the SA node and, consequently, the heart rate. The observed decrease in heart rate can be unambiguously interpreted as an increase in vagal activity. However, despite statistically proven changes in autonomic balance following cold water ingestion, its physiological manifestations were quite subtle in scope and, therefore, did not produce sharp variations in heart rhythm. Thus, our study results confirm previous findings regarding the reduction in heart rate associated with drinking cold water (Pereira et al., 2017; Girona et al., 2014). On the other hand, none of the participants included in our study exhibited vagus-induced bradycardia (<60 beats/min), unlike other studies that suggest that cold water ingestion activated the parasympathetic nervous system to such an extent that bradycardia developed (Routledge et al., 2002).

The power of low-frequency, which reflects the activity of both branches of the autonomic nervous system with a dominant sympathetic influence, increased significantly immediately after ingesting cold water. Its values remained nearly constant for the next 30 min, peaking at the 30th minute. This finding suggests that ingesting water below a certain intra-abdominal temperature triggers the sympathetic branch of the autonomic nervous system. This likely occurs as the body works to prevent heat loss through the skin, leading to noradrenaline-mediated vasoconstriction (Girona et al., 2014). Some studies have failed to show differences in LF values before and after ingesting cold water (Siddangoudra and Arjunwadekar, 2017), while others have reported a decrease in LF values immediately after drinking water at a temperature below body temperature (Pereira et al., 2017; Arjunwadekar and Siddangoudra, 2019). The discrepancies in LF values may stem from the fact that we tested all participants during the luteal phase, a period known for sympathetic dominance. In contrast, the authors of the studies we compared did not consider the menstrual cycle phase (Pereira et al., 2017), while in other studies, it was not mentioned whether the menstrual cycle phase was considered or not. Since LF serves as an indirect measure of sympathetic activity, our results align with studies that directly measured sympathetic effects through the skin by assessing peripheral vasoconstriction (Routledge et al., 2002; Scott et al., 2001). Scott et al. showed that after ingesting cold water, peripheral vasoconstriction increases and peaks at the 30th minute, which corresponds with our findings, showing that the mean LF value peaks between 30 and 35 min post-ingestion.

HF, pRR50%, and rMSSD are the best indicators of parasympathetic effects on heart rate. Therefore, the increase in these parameters, along with the decrease in heart rate, supports the idea that cold water ingestion activates the vagus nerve. The immediate increase in rMSSD, a more specific marker of parasympathetic activity due to its sensitivity to short-term beat-to-beat variability and independence from respiratory rate, suggests a rapid vagal response to cold water ingestion. In contrast, the delayed rise in HF power likely reflects its reliance on stable respiratory patterns and longer data windows for accurate spectral analysis, rather than a true delay in physiological vagal activation. This finding aligns with other studies confirming that drinking cold water boosts cardiac vagal tone (Pereira et al., 2017; Girona et al., 2014; Arjunwadekar and Siddangoudra, 2019). Both parasympathetic and sympathetic outputs show small fluctuations during the next half-hour after cold water ingestion, but these variations do not reach statistical significance. The parasympathetic system likely aims to prevent an excessive rise in blood pressure and heart rate, which could occur from solely activating the sympathetic branch of the autonomic nervous system.

In young, healthy subjects with preserved autonomic function, it is expected that both branches of the autonomic nervous system will work in harmony, resulting in proper heart rate control. The data we obtained in this study, through indirect measurements of autonomic function, suggest that ingesting cold water activates both the sympathetic and parasympathetic branches of the autonomic nervous system. A prerequisite for such a response of the body is the preserved function of both components of the autonomic nervous system. On the other hand, some studies have observed a transient increase in blood pressure in patients with inadequate ANS function, significantly longer than in the healthy population (Jordan et al., 1999; Jordan et al., 2001). This means that patients with comorbidities such as diabetic neuropathy, hypothyroidism, neurodegenerative diseases, or other conditions leading to autonomic dysfunction may experience a prolonged increase in blood pressure after ingesting cold water, along with all the damaging effects this can have on blood vessels. An inefficient vagal response to increased sympathetic output leads to this non-physiological phenomenon (Pereira et al., 2017; Girona et al., 2014; Routledge et al., 2002). Conversely, our results, in light of the decrease in heart rate after drinking cold water, could have a clinical benefit in patients who require a reduction in heart activity, such as those with acute myocardial infarction, but only if the function of the autonomic nervous system, particularly the vagal component, is preserved. Additionally, the increase in blood pressure caused by sympathetic activation following the consumption of cold water could be beneficial for patients with orthostatic hypotension.

One limitation of our study is that it only included young, healthy females in the luteal phase of the menstrual cycle. Since previous research suggests that autonomic tone varies throughout the menstrual cycle, future studies should explore whether similar responses occur in other phases. A larger population and stratification based on age, as well as the concurrent measurement of blood pressure, should be included in the future. Additionally, while short-term effects were assessed, the potential long-term adaptation of autonomic function to repeated cold water exposure remains unexplored and should be a focus of future research. Furthermore, future research should focus on the effect of cold water ingestion in patients whose comorbidities potentially lead to ANS dysfunction.

## Data Availability

The raw data supporting the conclusions of this article will be made available by the authors, without undue reservation.
